# Advances in nanomedicine drug delivery applications for HIV therapy

**DOI:** 10.4155/fsoa-2017-0069

**Published:** 2017-09-06

**Authors:** Paul Curley, Neill J Liptrott, Andrew Owen

**Affiliations:** 1 Department of Molecular & Clinical Pharmacology, Institute of Translational Medicine, University of Liverpool, Liverpool, UK; 2 European Nanomedicine Characterisation Laboratory, Department of Molecular & Clinical Pharmacology, Institute of Translational Medicine, University of Liverpool, Liverpool, UK

**Keywords:** biocompatibility, HIV, long-acting antiretroviral, nanomedicine

Despite significant advances in the treatment of human immunodeficiency virus (HIV), there remain challenges. HIV is a chronic disease and patient adherence to treatment is critical over a lifetime. Poor therapy adherence increases the likelihood of virological failure and emergence of resistant strains of HIV [1]. Poor aqueous drug solubility is a major limitation, negatively impacting oral bioavailability for many antiretroviral drugs [2]. Complete eradication resulting in cure has long been a focus of research efforts but the existence of cellular and anatomical regions where the virus can continue to replicate in subtherapeutic drug concentrations creates sanctuary sites [3], which reseed the blood when therapy is withdrawn [4]. The application of nanomedicine to current and future HIV therapeutic agents may offer bespoke solutions to the issues faced by traditionally formulated drugs. The purpose of this review is to highlight recent advances in the application of nanotechnology to drug delivery as summarized in Figure 1.

**Figure 1.**
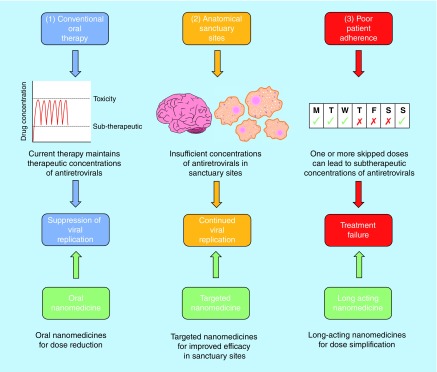
**Shows some of the limitations of current antiretroviral therapy and opportunities to address these limitations via nanomedicine.** **(1)** Conventional oral nanomedicine is effective at achieving therapeutic concentrations of antiretrovirals and maintaining suppression of viral replication. However, application of oral nanomedicine offers potential benefits such as dose reduction, reduced toxicity and improved bioavailability. **(2)** Subtherapeutic concentrations of antiretrovirals in sanctuary sites allow continued viral replication in key anatomical and cellular regions despite viral suppression in the plasma. Targeted nanomedicines offer the opportunity to deliver therapeutic concentrations of antiretrovirals to sanctuary sites. **(3)** Poor patient adherence may produce subtherapeutic concentrations of antiretrovirals which may lead to viral rebound. Application of long-acting nanomedicines may mitigate the risk effects of poor patient compliance.

## Oral nanomedicines

A number of nanomedicine strategies are being explored for oral delivery of antiretroviral drugs and have been reviewed previously [5]. Among the current strategies to improve bioavailability of poorly water-soluble drugs are solid drug nanoparticle (SDN) formulations. SDNs of efavirenz were recently generated using a single-step emulsion-templated freeze-drying technique [6]. *In vitro–in vivo* pharmacokinetic extrapolation predicted that the SDN formulations could achieve similar pharmacokinetics to the standard Sustiva^©^ formulation with a 50% lower dose. These simulations were validated *in vivo* with pharmacokinetics in rats demonstrating a higher C_max_, C_min_ and AUC compared with a conventional preclinical formulation [6].

Currently, the ritonavir-boosted lopinavir (Kaletra^©^) pediatric formulation consists of an oral solution of 42% ethanol and 15% propylene glycol [7], which is clearly undesirable. This has spurred efforts to develop other formats (such as ‘sprinkles’) that despite some success have proven challenging due to issues such as taste masking. Recently, lopinavir SDN formulations (alone and in combination with ritonavir) were produced with potential for an ethanol-free pediatric liquid formulation. *In vivo* pharmacokinetics demonstrated similar plasma concentrations to a conventional lopinavir preclinical formulation, without the need for an organic solvent [7].

Unlike many nanomedicine strategies, the SDN technologies described above are translatable to industrial scale through spray-dry manufacture and they utilize already licensed drugs in combination with US FDA Center for Drug Evaluation and Research (CDER) listed excipients. Thus, they potentially provide low-cost solutions to free up manufacturing capacity, while reducing doses required for effective therapy [7]. Importantly however, other advanced strategies involving lipid-based or polymer-based nanocarrier systems have also shown preclinical success in augmenting oral delivery [8–10]. It should be noted that many of these strategies involve carrier materials (or excipients) that are in themselves novel, which may present hurdles to translation, necessitating additional safety assessments.

## Long-acting injectable nanomedicines

Problems with low adherence are currently being addressed by development of long-acting injectable (LAI) formulations [2]. The non-nucleoside reverse transcriptase inhibitor rilpivirine has been available for oral administration since 2011. Recently, rilpivirine has been nanoformulated to produce an LAI. When administered subcutaneously or intramuscularly, rilpivirine concentrations were detectable for up to 2 months in rats and 6 months in dogs [11]. This was followed up by successful assessment in 51 healthy volunteers where rilpivirine plasma concentrations remained above 10 ng/ml for up to 26 weeks with no grade 3 or 4 adverse events [12].

HIV therapy is most successful when a combination of drugs are administered. Cabotegravir (an integrase inhibitor) is currently being developed for application for both oral and LAI purposes [13]. In a recent study involving 56 healthy volunteers, cabotegravir LAI (administered either as intramuscular or subcutaneous injection) sustained exposure for up to 24 weeks. Similar to rilpivirine LAI, cabotegravir was well tolerated with no grade 2–4 injection site reactions reported [14].

The rilpivirine and cabotegravir LAI formulations are showing great promise for treatment and pre-exposure prophylaxis for HIV. Notwithstanding, current paradigms for HIV treatment necessitate the combination of therapeutic agents. Accordingly, Janssen and ViiV Healthcare have partnered to evaluate the utility of cabotegravir and rilpivirine coadministration as a combination LAI therapy. Recent data from the LATTE II study have been extremely encouraging in support of this combination as a maintenance therapy [15], with this strategy advancing via separate intramuscular depots for each drug rather than a combination product.

Recently, a combination (tenofovir and lopinavir/ritonavir) lipid-stabilized nanosuspension was administered subcutaneously to macaques. Encouragingly, tenofovir plasma and peripheral blood mononuclear cell concentrations were detectable over 2 weeks. Peripheral blood mononuclear cell exposure of total tenofovir and tenofovir-diphosphate was markedly higher in comparison to conventional oral tenofovir disoproxil fumarate or tenofovir alafenamide [16].

## Targeted nanomedicines

As mentioned previously, a significant barrier to HIV eradication is the presence of sanctuary sites. For example, the central nervous system (CNS) represents an anatomical sanctuary site for many drugs. To target delivery to the brain, magnetic azidothymidine 5′-triphosphate liposomal nanoformulations have been developed. In an *in vitro* model of the blood–brain barrier, the transcellular apparent permeability was increased (when a magnetic field was applied) versus a free drug [17].

Macrophages also represent an important target in HIV. Through targeting of the mannose receptor, nanocarrier systems have been proposed that may be suitable for HIV therapy. By optimizing the number of mannose units, polyethylene glycol chain-length and spacer-length, mannosylated polyethylene glycol conjugate nanocarriers were generated. *In vitro* uptake by J774.E murine macrophage-like cells was confirmed using confocal microscopy and the optimal configuration was identified as: small polyethylene glycol polymer carrier, two mannose units per nanocarrier and 56 Å distance between mannose units [18].

These data support further research into strategies to increase delivery to the CNS, lymph or cellular reservoirs. However, the complexity of these systems complicates compatibility with patient acceptable routes of administration (often requiring repeated intravenous delivery) and cost-effective routes to scalable manufacture. Also, translation clearly demands thorough investigation of the potential safety concerns arising from the altered distribution and in some cases the additional complexity introduced by the need for additional noninvasive equipment and medical care. Many of these issues are exacerbated by the need to develop systems for implementation in low-income and middle-income countries where the overwhelming burden of disease resides.

## Safety

It is becoming increasingly apparent that some nanoparticle platform technologies have a propensity for interaction with the immune system. Therefore, for complex multicomponent nanocarrier systems, a thorough analysis of potential interactions is required, in addition to a robust understanding of the implication for altered distribution. Conventional drug formulations are often too small to interact with the immune system whereas nanoparticles are in the size range and have physical characteristics that may be recognized by immune cells with associated implications for safety [19]. As such, the assessment of biocompatibility of nanoformulations is an increasingly important area of research [20].

The safety and biocompatibility testing of nanoparticles represents a significant challenge driven in part by the huge diversity of materials. Particles range in size (1–1000 nm), functionality, charge and composition [21]. This diversity means that existing immunological assays are sometimes inadequate to account for nanoparticle characteristics [22]. For example, some nanoparticles possess catalytic properties which interfere with assays meaning careful scrutiny is needed to ensure validity of *in vitro* toxicity tests [19].

Many nanomedicines aim to alter the pharmacokinetics or distribution. Thus, there is a need to better understand the exposure–response relationship, particularly for nanomedicines that interact with the immune system. In case of HIV infection, greater accumulation within macrophages may have a therapeutic benefit as a sanctuary site for the virus. However, increasing exposure to macrophages may exacerbate the impact on cell function thereby necessitating careful understanding of what is required pharmacologically as well as in terms of safety.

## Discussion & future perspective

The application of nanomedicine to HIV presents many exciting opportunities. It is well known that during the development of a new therapeutic, many candidates will fail to reach the clinic. Frequently reported reasons for failure in Phase I and Phase II trials are toxicity, inadequate efficacy and poor pharmacokinetics [23]. Even when medicines transition to commercial applications, careful post-licensing optimization is often required. Nanotechnology offers the opportunity to develop strategies for addressing the limitations of current, future and even failed therapeutics. Importantly, a major driver for nanomedicine development has been the improvement of safety by reducing doses or improving distribution to diseased cells.

Despite increasing interest in nanomedicines, there are still significant gaps in knowledge of the underlying mechanisms. For example, a greater understanding of drug release following LAI injection is needed [2]. Recent work on similar SDN formulations of paliperidone palmitate has demonstrated the importance of granuloma and phagocytosis by infiltrating macrophages [24]. Further support for the role of macrophages has been demonstrated in rodents, where nanoparticle-mediated activation of autophagy led to 50-fold increase in the plasma concentration of the viral integrase inhibitor dolutegravir [25]. Such information on nanospecific drug metabolism and pharmacokinetics will be central to bringing forward the optimized next generation of LAI medicines.

## Conclusion

In summary, a better understanding of the mechanisms underpinning nanoparticle behavior will enable rational design of nanomedicines with greater efficacy and improved safety so that the preclinical promises can be realized for patients, across the different nanotechnologies being explored.
